# Supplementary material for: Response outcome gates the effect of spontaneous cortical state fluctuations on perceptual decisions
Source: eLife. 2023 May 17;12:e81774. doi: 10.7554/eLife.81774 (PMC10241512; doi:10.7554/eLife.81774)
Supplement: Supplementary file 1. — Starting from the left, each column represents: the figure in the text where the results are displayed, the prediction target, the predictors (one row per predictor), the median and lower and upper limits of the 95% confidence interval (Methods), the associated bootstrap p value (Methods), and the total number of observations (number of rows in the predictor matrix) in the model. [file elife-81774-supp1.pdf]

Figure	Target	Predictor	Median	95% CI_{Low}	95% CI_{High}	p-value	N_{obs}
2A	FR	Intercept	1.66	1.65	1.68	<0.0002	5687
		OpticF	0.06	0.04	0.08	<0.0002	
		PupilS	0.04	0.02	0.05	0.67	
2A	Synch	Intercept	1.095	1.092	1.099	<0.0002	5687
		OpticF	-0.0007	-0.0041	0.0027	0.72	
		PupilS	0.0029	-0.0004	0.0060	0.09	
2D	FR	Intercept	0.004	-0.006	0.015	0.40	5687
		$OpticF_I$	0.091	0.078	0.106	<0.0002	
		$PupilS_I$	0.004	-0.007	0.015	0.52	
2D	Synch	Intercept	0.0004	-0.0023	0.0030	0.77	5687
		$OpticF_I$	0.004	0.001	0.007	0.012	
		$PupilS_I$	-0.008	-0.011	-0.005	<0.0002	
3A	Accuracy	Intercept	1.20	1.09	1.31	<0.0002	3230
		Stim	0.60	0.46	0.76	<0.0002	
		pCorr	-0.13	-0.23	-0.04	0.006	
		TrN	0.14	0.04	0.24	0.005	
		$OpticF_I$	0.01	-0.09	0.12	0.83	
		$PupilS_I$	0.009	-0.090	0.111	0.85	
		FR_I	0.07	-0.02	0.16	0.16	
		$Synch_I$	-0.005	-0.010	0.091	0.91	
3C	Accuracy	Intercept	1.49	1.25	1.77	<0.0002	757
		Stim	0.75	0.44	1.15	<0.0002	
		TrN	0.33	0.11	0.57	0.005	
		$OpticF_I$	0.01	-0.21	0.25	0.92	
		$PupilS_I$	0.01	-0.20	0.24	0.90	
		FR_I	0.28	0.07	0.49	0.006	
		$Synch_I$	-0.28	-0.50	-0.060	0.012	
		FR_I & $Desynch_I$	0.36	0.15	0.58	0.0006	
3E	Accuracy	Intercept	1.15	1.03	1.28	<0.0002	2473
		Stim	0.60	0.45	0.79	<0.0002	
		TrN	0.12	0.01	0.23	0.03	
		$OpticF_I$	0.001	-0.119	0.125	0.98	
		$PupilS_I$	0.004	-0.111	0.121	0.95	
		FR_I	0.03	-0.08	0.14	0.64	
		$Synch_I$	0.07	-0.04	0.18	0.22	
		FR_I & $Synch_I$	0.07	-0.04	0.18	0.20	
3H	Accuracy	Intercept	1.58	1.34	1.87	<0.0002	833
		Stim	0.64	0.35	1.05	<0.0002	
		TrN	0.29	0.09	0.53	0.007	
		$OpticF_I$	-0.06	-0.30	0.19	0.59	
		$PupilS_I$	-0.18	-0.41	0.04	0.11	
		FR_I	0.04	-0.18	0.27	0.70	
		$Synch_I$	-0.01	-0.24	0.21	0.92	
3I	Accuracy	Intercept	1.33	1.20	1.47	<0.0002	2400
		Stim	0.54	0.40	0.72	<0.0002	
		TrN	0.20	0.09	0.32	0.001	
		$OpticF_I$	-0.04	-0.16	0.09	0.57	
		$PupilS_I$	0.06	-0.06	0.18	0.30	
		FR_I	-0.004	-0.113	0.105	0.95	
		$Synch_I$	0.08	-0.03	0.20	0.14	
4E	pCorr	Intercept	1.63	1.48	1.80	<0.0002	3230
		TrN	0.25	0.14	0.36	0.001	
		$OpticF_I$	0.08	-0.05	0.21	0.24	
		$PupilS_I$	1.18	1.02	1.35	<0.0002	
		FR_I	-0.22	-0.34	-0.10	<0.0002	
		$Synch_I$	-0.0002	-0.1052	0.1056	0.995	
5D	Premature	Intercept	-2.82	-3.01	-2.64	<0.0002	4634
		TrN	-0.36	-0.51	-0.22	<0.0002	
		pPrem	-0.04	-0.24	0.09	0.56	
		pCorr	0.17	0.02	0.35	0.03	
		pSkip	-0.08	-0.29	0.07	0.30	
		$OpticF_I$	0.14	0.01	0.27	0.03	
		$PupilS_I$	-0.17	-0.32	-0.03	0.02	
		FR_I	0.03	-0.10	0.16	0.60	
		$Synch_I$	0.11	-0.02	0.23	0.09	
5F	Skip	Intercept	-1.74	-1.94	-1.59	<0.0002	5488
		TrN	0.65	0.52	0.81	<0.0002	
		pPrem	-0.09	-0.22	0.01	0.08	
		pCorr	-0.38	-0.50	-0.25	<0.0002	
		pSkip	0.54	0.44	0.64	<0.0002	
		$OpticF_I$	0.21	0.10	0.33	0.0006	
		$PupilS_I$	0.04	-0.06	0.14	0.40	
		FR_I	0.07	-0.03	0.16	0.19	
		$Synch_I$	0.005	-0.084	0.098	0.90	
